# Microstructure and Mechanical Properties of High-Entropy Alloy Co_20_Cr_26_Fe_20_Mn_20_Ni_14_ Processed by High-Pressure Torsion at 77 K and 300 K

**DOI:** 10.1038/s41598-018-29446-y

**Published:** 2018-07-23

**Authors:** Jongun Moon, Yuanshen Qi, Elena Tabachnikova, Yuri Estrin, Won-Mi Choi, Soo-Hyun Joo, Byeong-Joo Lee, Aleksey Podolskiy, Mikhail Tikhonovsky, Hyoung Seop Kim

**Affiliations:** 1 Department of Materials Science and Engineering, Pohang University of Science and Technology (POSTECH), Pohang, 790-784 Korea; 2 Center for High Entropy Alloys, Pohang University of Science and Technology (POSTECH), Pohang, 790-784 Korea; 3 Department of Materials Science and Engineering, Technion – Israel Institute of Technology, 32000 Haifa, Israel; 4 B. Verkin Institute for Low Temperature Physics and Engineering of National Academy of Sciences of Ukraine, 47 Nauky Ave., Kharkov, 61103 Ukraine; 5 Department of Materials Science and Engineering, Monash University, Melbourne, VIC 3800 Australia; 6 Department of Mechanical Engineering, The University of Western Australia, Crawley, WA6009 Australia; 7 Institute of Materials Research, Tohoku University, Sendai, 980-8577 Japan; 8 National Science Center Kharkov Institute of Physics and Technology of National Academy of Sciences of Ukraine, 1 Academicheskaya street, Kharkov, 61108 Ukraine

## Abstract

In this work, the mechanical characteristics of high-entropy alloy Co_20_Cr_26_Fe_20_Mn_20_Ni_14_ with low-stacking fault energy processed by cryogenic and room temperature high-pressure torsion (HPT) were studied. X-ray diffraction, scanning electron microscopy (SEM), and transmission electron microscopy (TEM) analyses were performed to identify the phase and microstructure variation and the mechanical properties characterized by Vickers hardness measurements and tensile testing. Cryogenic HPT was found to result in a lower mechanical strength of alloy Co_20_Cr_26_Fe_20_Mn_20_Ni_14_ than room temperature HPT. Microstructure analysis by SEM and TEM was conducted to shed light on the microstructural changes in the alloy Co_20_Cr_26_Fe_20_Mn_20_Ni_14_ caused by HPT processing. Electron microscopy data provided evidence of a deformation-induced phase transformation in the alloy processed by cryogenic HPT. Unusual softening phenomena induced by cryogenic HPT were characterized by analyzing the dislocation density as determined from X-Ray diffraction peak broadening.

## Introduction

High-entropy alloys (HEAs) composed of five or more principal elements with an elemental concentration of 5 to 35 at% each are novel materials with a simple single phase face-centered cubic (FCC), body-centered cubic (BCC), or hexagonal close-packed (HCP) structure^1–3^. Specifically, FCC alloy CoCrFeMnNi, also known as the Cantor alloy, has outstanding mechanical properties at low temperatures^4,5^ and the extensive research of the alloy was undergone so far^6–13^. The observed enhancement of strength and tensile ductility at cryogenic temperatures is largely attributed to high activity of twinning owing to a low stacking fault energy (SFE) of the alloy^14^, which is a common feature of HEAs^5,15^.

Since the inception of the concept of HEAs, numerous studies have been conducted to characterize their mechanical properties and microstructure evolution, chiefly of coarse-grained HEAs^16–18^. Recently, efforts to study ultrafine-grained (UFG) HEAs produced by severe plastic deformation (SPD) have been undertaken^19–22^. HEAs with nano or sub micron-grained structure have been shown to result in excellent mechanical properties^13,14^. In addition, since more conventional, coarse-grained HEAs exhibit a good combination of high strength and high fracture toughness at low temperatures^4,23^, an even better property profile can be expected of HEAs processed by SPD at cryogenic temperatures. High-pressure torsion (HPT) is one of the most popular SPD techniques as it is very efficient in reducing the grain size through a combination of high pressure and giant shear strain^24–26^. Previously, nanocrystalline states were produced in a single phase CoCrFeMnNi HEA using HPT at ambient temperature^22^. It was shown by tensile testing^22^ that a transition to a nanocrystalline structure gives rise to a significant increase of the room temperature strength accompanied with moderate ductility. Additional improvement of mechanical characteristics was achieved by decreasing the temperature of HPT to the cryogenic region^27^.

It is therefore of great interest to produce a nanocrystalline or UFG structure in the CoCrFeMnNi HEA by cryogenic HPT (‘cryo-HPT’) and to study the effect of the processing temperature on its microstructure and the mechanical properties. In our previous study^28^, the deformation-induced FCC-to-HCP phase transformation of the Co_20_Cr_26_Fe_20_Mn_20_Ni_14_ alloy with low-SFE (3.5 mJ/m^2^)^15^ processed by cryo-HPT was investigated. The present follow-up work investigates the microstructure and mechanical properties of the Co_20_Cr_26_Fe_20_Mn_20_Ni_14_ alloy processed by HPT at both liquid nitrogen temperature and room temperature have been investigated. The results of this in-depth investigation are reported below.

## Results

### Vickers hardness and tensile properties

**Figure 1:**
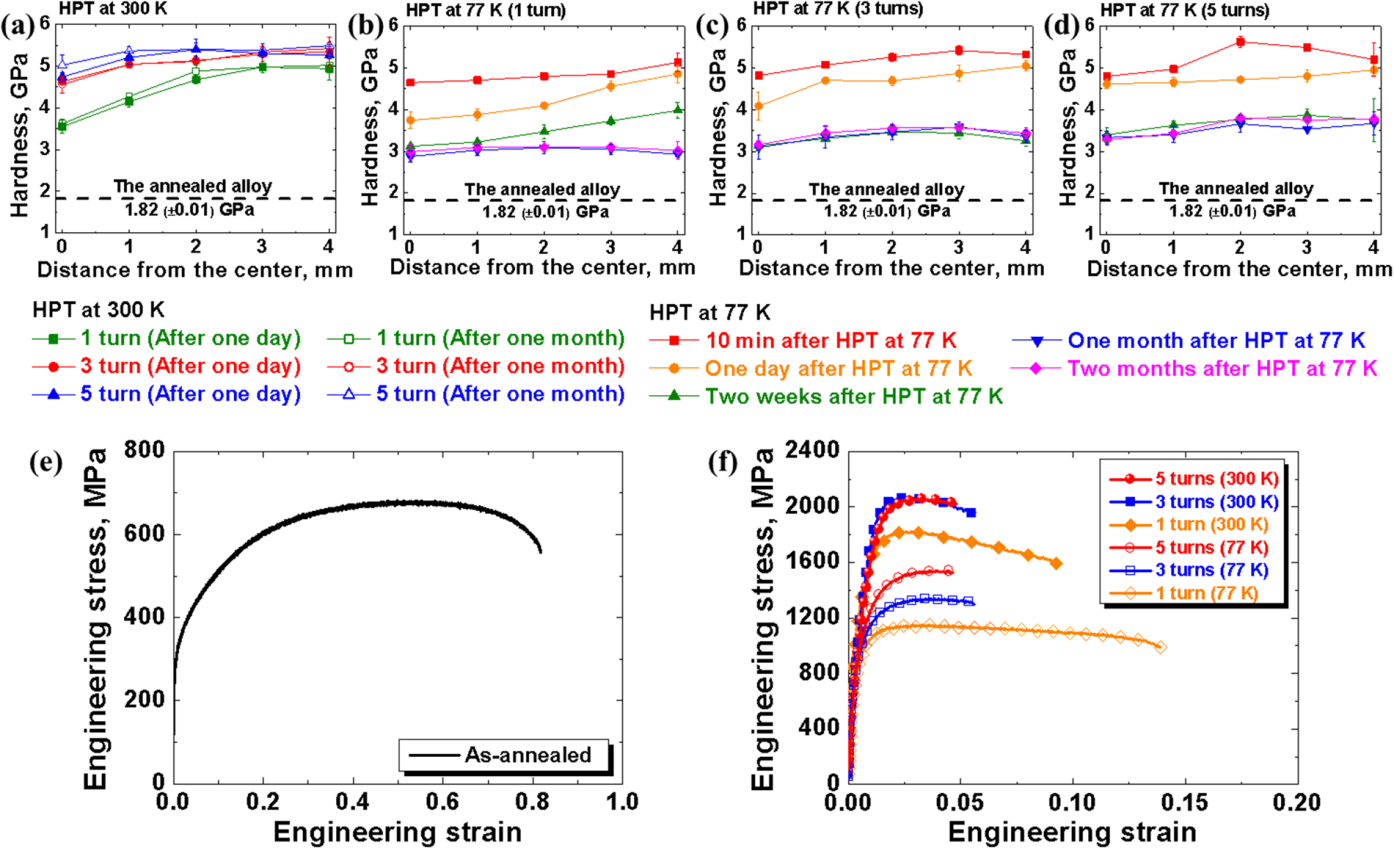
Mechanical properties of Co_20_Cr_26_Fe_20_Mn_20_Ni_14_ alloy after the HPT processing at 77 K and 300 K. Variation of Vickers hardness with the distance from the center of the HPT sample (**a**) one day, one month after HPT processing of Co_20_Cr_26_Fe_20_Mn_20_Ni_14_ alloy at 300 K and (**b**–**d**) 10 minutes, one day, two weeks, one month and two months after HPT process at 77 K. Room temperature stress-strain curves for Co_20_Cr_26_Fe_20_Mn_20_Ni_14_ alloy: (**e**) in the initial annealed state; (**f**) two weeks after the HPT processing at 77 K and 300 K.

Figure 1(a–d) display the variation of the Vickers hardness at the surface of the disc, from the center to the edge after various holding time after HPT. The applied shear strain is calculated as $\frac{2\pi Nr}{h}$, where *N* is the number of anvil turns, *r* is the distance from the axis of the HPT sample, and *h* is its thickness. Thus, the applied shear strain increases with the distance from the center of the disc. As seen from these diagrams, there is a general trend for the hardness to increase with shear strain and to saturate at large strains. Figure 1(a) indicates the measured Vickers hardness values one day and one month after HPT at room temperature. As the holding time after HPT at room temperature increased from one day to one month, a decrease in Vickers hardness was not observed. This ensures the stability of microstructure and mechanical properties after HPT processing at room temperature.

The evolution of hardness with ‘natural aging’ time after cryo-HPT was also monitored. It were presented in Fig. 1(b–d), showing the Vickers hardness measured from the center to the edge after 10 minutes, one day, two weeks, one month, and two months following HPT by 1, 3, and 5 anvil turns at 77 K. An appreciable drop in hardness with the natural aging time is seen. It is evident from Fig. 1(b–d) that the Vickers hardness 10 minutes after the cryo-HPT (at 77 K) was originally higher than that for the room temperature HPT (at 300 K) and eventually kept decreasing with natural aging time. Notably, the drop after two weeks following cryo-HPT was precipitous, followed by a less drastic decrement after one month of natural aging. Vickers hardness stabilized then, and continued natural aging beyond this time did not lead to its further decrease. As a matter of fact, the saturation hardness after cryo-HPT was higher than that of the as-annealed alloy.

The results of tensile testing for the annealed and the HPT-processed Co_20_Cr_26_Fe_20_Mn_20_Ni_14_ alloy conducted two weeks after HPT processing are presented in Fig. 1(e,f) and Supplementary Table S1. The lower tensile strength of cryo-HPT processed material compared to that pre-deformed by room temperature HPT supports the Vickers hardness data. A slightly higher tensile elongation of the cryo-HPT processed specimen is also noted. A further observation to be mentioned is that the specimens that experienced the largest shear strains under cryo-HPT, i.e. those deformed by five turns of the anvil, exhibited the least drops of hardness after two weeks of natural aging.

### Microstructure evolution of the alloy after HPT at 300 K and 77 K

**Figure 2:**
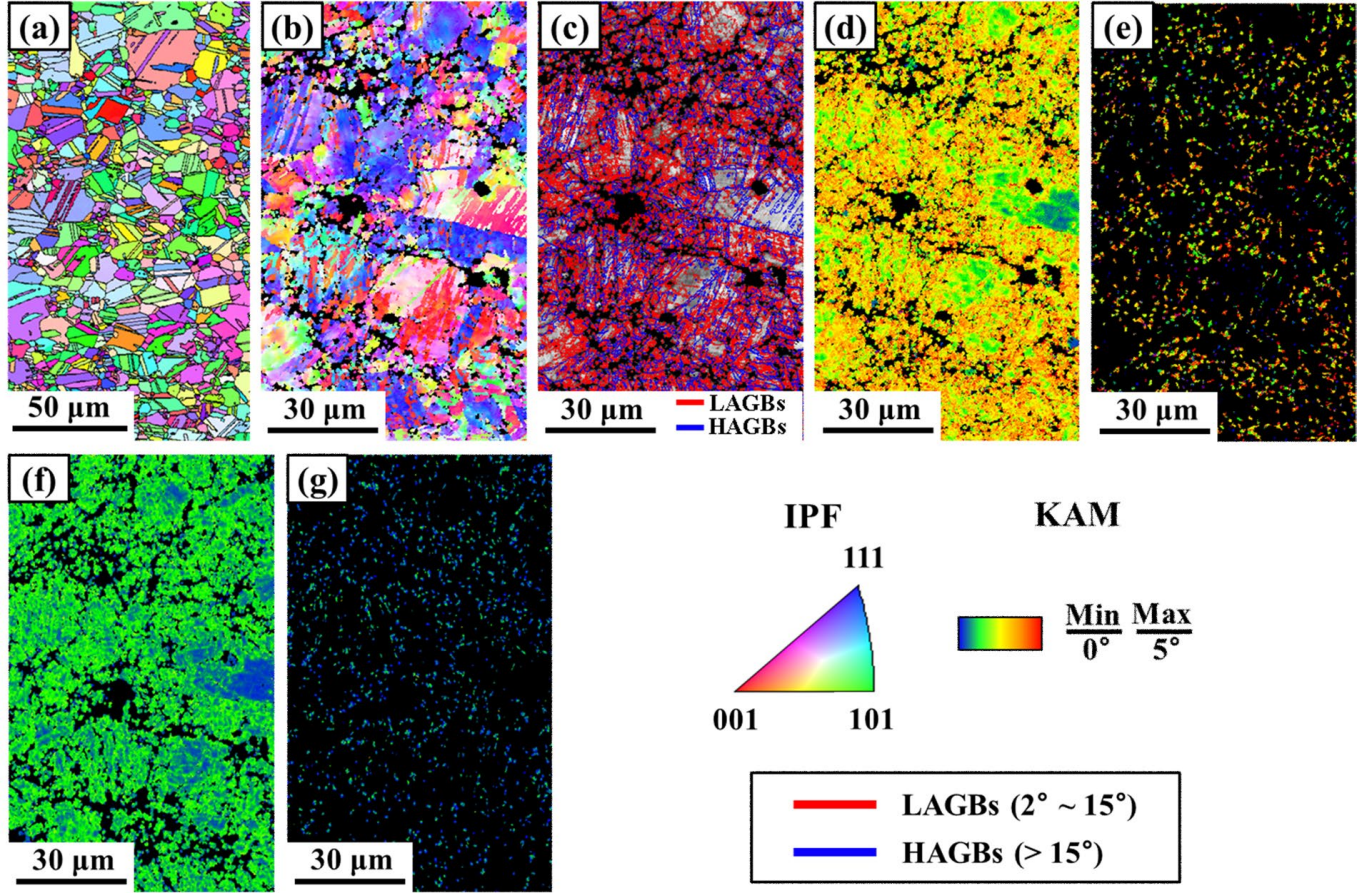
Microstructural evolution of Co_20_Cr_26_Fe_20_Mn_20_Ni_14_ alloy with natural aging time after 5 turns of HPT at 77 K. (**a**) IPF map of the annealed Co_20_Cr_26_Fe_20_Mn_20_Ni_14_ alloy. (**b**) IPF, (**c**) grain boundary map, and (**d**,**e**) KAM maps two hours after HPT processing at 77 K, (**f**,**g**) KAM maps for the material naturally aged for two weeks after HPT processing at 77 K. Note: (**e**,**g**) show the grains with a size smaller than 1 µm.

Figure 2(a) shows the inverse pole figure (IPF) map of the annealed Co_20_Cr_26_Fe_20_Mn_20_Ni_14_ alloy obtained by Electron backscattered diffraction (EBSD) analysis. The average grain size in this condition was calculated to be 31.8 ± 15.9 µm.

The microstructures observed two hours and two weeks after cryo-HPT processing were analysed to reveal the microstructure evolution with natural aging. Figure 2(b–g) shows an IPF image, a grain boundary map, and kernel average misorientation (KAM) maps. Figure 2(b–e) were obtained two hours after cryo-HPT. Coarse grains with a large amount of low angle grain boundaries (LAGBs) were observed in Fig. 2(b,c). In Fig. 2(d,e), the KAM was calculated up to the third-nearest neighbour shell with a maximum misorientation angle of 5°^11^. Most of the grains, including the grains below 1 μm in size, exhibit large KAM values, indicating a high density of geometrically necessary dislocations due to dislocation rearrangement. However, the KAM maps for the material studied after two hours and two weeks of natural aging following cryo-HPT processing were entirely different. Figure 2(f,g) show the KAM maps taken after two weeks following HPT. The KAM values were significantly lower, especially for grains under 1 μm in size for which they dropped to nearly zero.

**Figure 3:**
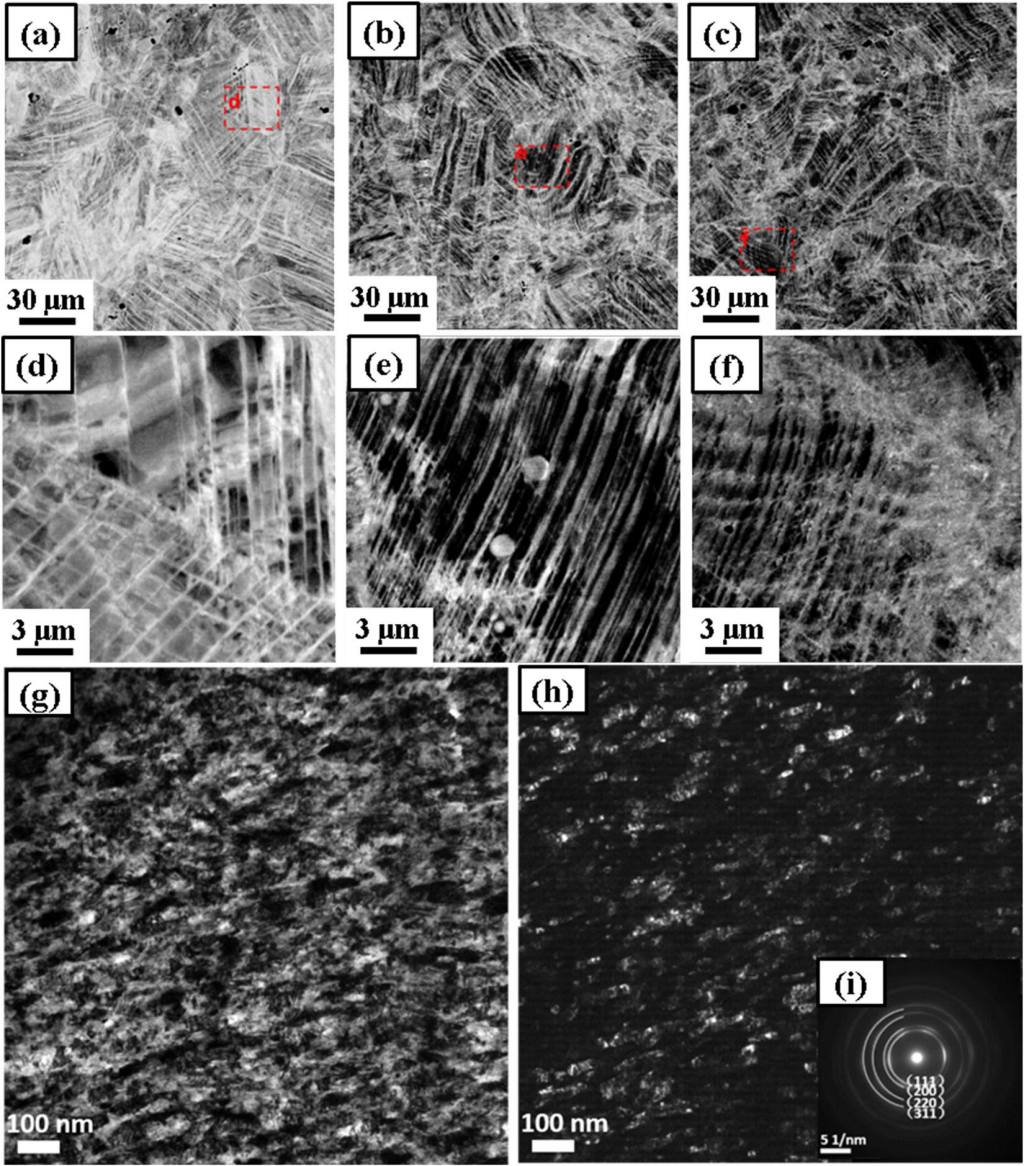
Microstructures of Co_20_Cr_26_Fe_20_Mn_20_Ni_14_ alloy after HPT at 77 K and 300 K. SEM-BSE images of Co_20_Cr_26_Fe_20_Mn_20_Ni_14_ alloy after (**a**,**d**) 1 turn, (**b**,**e**) 3 turns, and (**c**,**f**) 5 turns of cryo-HPT at 77 K. (**d**–**f**) are the magnified images of the corresponding areas highlighted in (**a**–**c**). TEM images of the Co_20_Cr_26_Fe_20_Mn_20_Ni_14_ alloy after 5 turns of HPT at 300 K (**g**) bright field image, (**h**) tilted dark field image, and (**i**) inset showing the corresponding SADP.

Figure 3(a–f) show scanning electron microscopy-backscattered electron (SEM-BSE) images of Co_20_Cr_26_Fe_20_Mn_20_Ni_14_ alloy after 1 turn, 3 turns, and 5 turns of cryo-HPT at 77 K, respectively. The activation of the slip systems with planar glide of dislocations and the formation of a dislocation Taylor lattice with features of a cellular structure after the first HPT turn is observed in Fig. 3(a,d). With an increasing number of turns, the density of slip traces increased and their spacing decreased significantly to accommodate the strain imposed by HPT. Furthermore, as shown in the high magnification images of Fig. 3(d–f), the arrays evolve from straight to curved ones. This suggests that further slip systems and microbands were activated with increasing number of HPT revolutions.

Figure 3(g,h) show, respectively, transmission electron microscopy (TEM) bright field (BF) and dark field (DF) micrographs of the alloy processed by five turns of HPT at 300 K. The average grain size was calculated to be 61 ± 25 nm, indicating extreme grain refinement down to nanometer scale. In the BF image of Fig. 3(g), grain boundaries are not well-defined, which suggests that dynamic recrystallization was not fully developed. From Fig. 3(h), it is seen that the grains are elongated in the shearing direction. The continuous ring pattern in Fig. 3(i) indicates that the nano-grains have random orientations.

**Figure 4:**
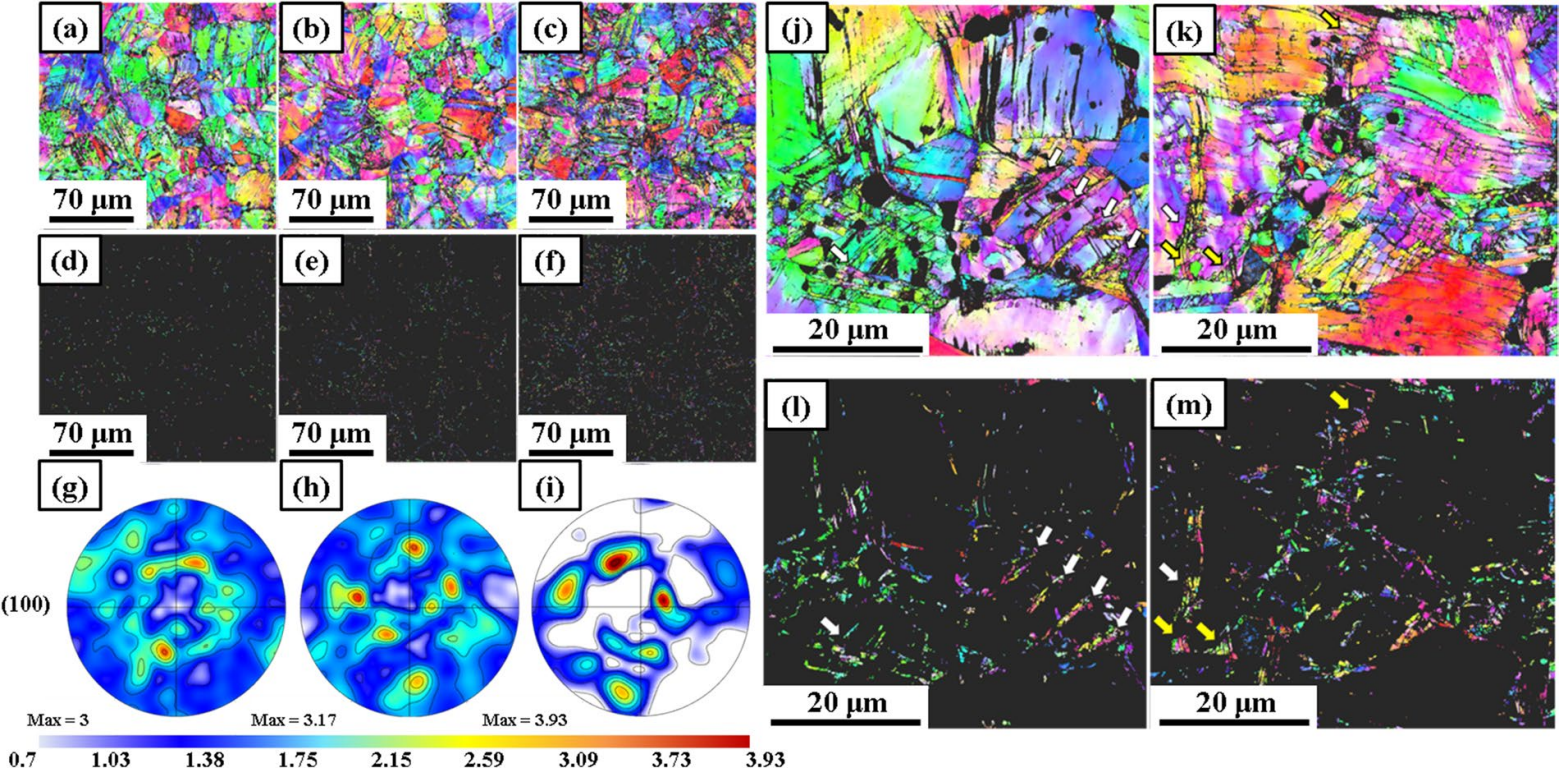
Evolution of microstructures and the corresponding textures of Co_20_Cr_26_Fe_20_Mn_20_Ni_14_ alloy with the number of cryo-HPT turns. IPF images and (100) pole figures of (**a**,**d**,**g**) 1 turn, (**b**,**e**,**h**) 3 turns, and (**c**,**f**,**k**) 5 turns. Note: (**d**–**f**) show the grains with size smaller than 1 µm (labeled GS < 1 µm). The maximum texture intensity is indicated for each pole figure in the units of multiple random distribution (mrd). IPF images of Co_20_Cr_26_Fe_20_Mn_20_Ni_14_ alloy after (**j**,**l**) 1 turn and (**k**,**m**) 5 turns of HPT at 77 K. Note: (**l**,**m**) show the grains with a size smaller than 1 µm.

Figure 4(a–f) show inverse pole figures of the alloy after 1, 3, and 5 turn(s) of cryogenic HPT, respectively. Similar to Fig. 3(a–c), no significant microstructure evolution with the number of cryo-HPT turns is discernible in Fig. 4(a–c). However, when inspecting the grains whose size is smaller than 1 µm in Fig. 4(d–f) one can see that the total area of such grains increased five-fold as the sample went from 1 turn to 3 turns and was further doubled when the sample went from 3 to 5 cryo-HPT turns. The average grain size dropped from 27.53 ± 3.37 μm (the value it had after 1 turn) to 13.59 ± 1.93 μm and 5.42 ± 1.69 μm after 3 and 5 turns, respectively. The maximum texture intensity increased with the number of anvil revolutions from 3 mrd (multiple of random distribution) after 1 turn to 3.17 and 3.93 mrd after 3 and 5 turns (Fig. 3(g–i)). Another observation of note is that the volume fraction of twins was rather insignificant. The deformation twin volume fraction was calculated based on the EBSD results. The measured area of the identified deformation twins was divided by the total measured area. The twin volume fraction of annealed Co_20_Cr_26_Fe_20_Mn_20_Ni_14_ alloy was around 48%; it decreased with the number of cryo-HPT turns down to 2.3%, 1.4%, and 0.6% after 1, 3, and 5 turns, respectively.

Deformation-induced and/or annealing twins were also found to contribute to grain refinement. Indeed, a close inspection of Fig. 4(j,l) shows that after one HPT turn at 77 K, strain-induced fragmentation of twins gave rise to grain refinement, as indicated by the white arrows. Fragmentation of shear bands is yet another mechanism of grain refinement, which is apparent for higher strains. It is indicated by the white arrows in Fig. 4(k,m). Some ultrafine grains located at grain boundaries of the severely bent coarse grains, marked by the yellow arrows, are also discernible in Fig. 4(k,m). This could be caused by the interaction between the planar dislocations or shear bands and grain boundaries.

**Figure 5:**
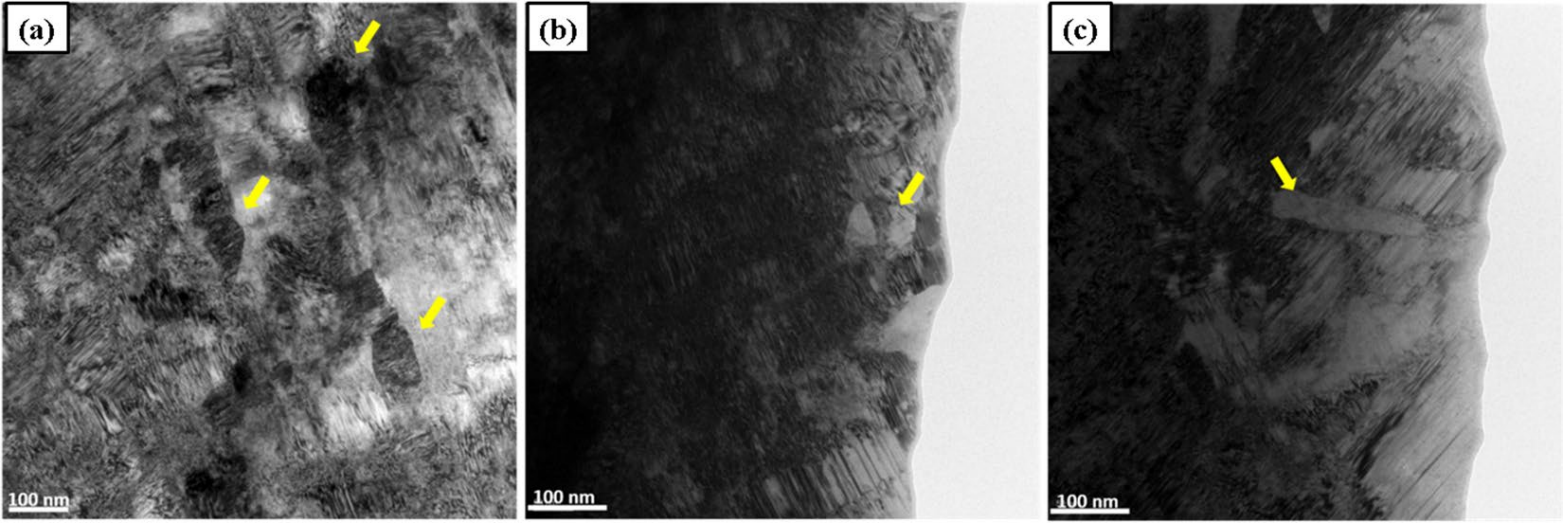
TEM BF images of Co_20_Cr_26_Fe_20_Mn_20_Ni_14_ alloy after 5 turns of HPT at 77 K. (**a**–**c**) Typical nano-sized grains, lamellar twin structures, and coplanar slip traces in the Co_20_Cr_26_Fe_20_Mn_20_Ni_14_ alloy after 5 turns of HPT at 77 K.

The nano scale microstructural features of the sample that went through 5 HPT turns at 77 K are presented in high-magnification TEM BF images in Fig. 5. Equiaxed grains with a size around 100 nm are marked by the yellow arrows in Fig. 5(a,b). A microband exhibiting a slip system that is different from those operating outside of the microband on either side is highlighed by a yellow arrow in Fig. 5(c).

**Figure 6:**
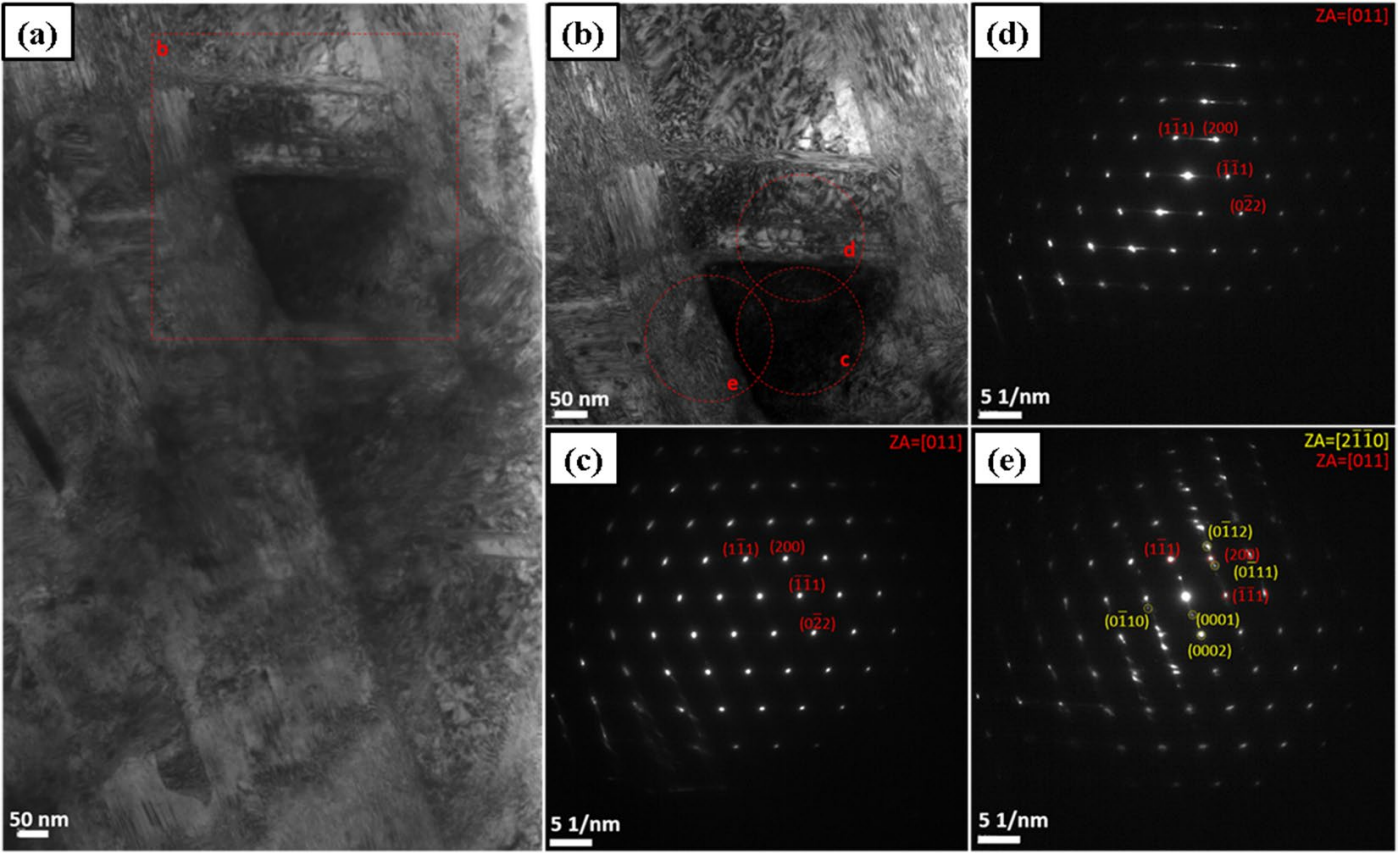
TEM micrographs of the alloy deformed by 5 HPT turns at 77 K. (**a**,**b**) BF images and (**c**–**e**) SADPs showing grain subdivision and a phase transformation from FCC to HCP. The red dashed circles in (**b**) show where the SADPs presented in (**c**–**e**) were taken.

Figure 6(a,b) present an ultrafine grain as an evidence of subdivision of a microband during cryo-HPT. It can be seen that the active slip system in the microband is different from the ones operating in the bulk of the material on either side of it. A high density of dislocations tangled within the band is also seen. The ultrafine grain was tilted to its [011] zone axis and its corresponding selected area diffraction pattern is shown in Fig. 6(c). The selected area diffraction pattern (SADP) in Fig. 6(d) was taken slightly above that in Fig. 6(c). The diffuse streaks in that pattern indicate a continual lattice rotation originating from the planar glide of dislocations. However, in Fig. 6(e), instead of the pattern for FCC zone axis [011], that of the zone HCP axis [$2\bar{1}\bar{1}0$] is detectable.

### X-ray diffraction results and dislocation density evolution

**Figure 7:**
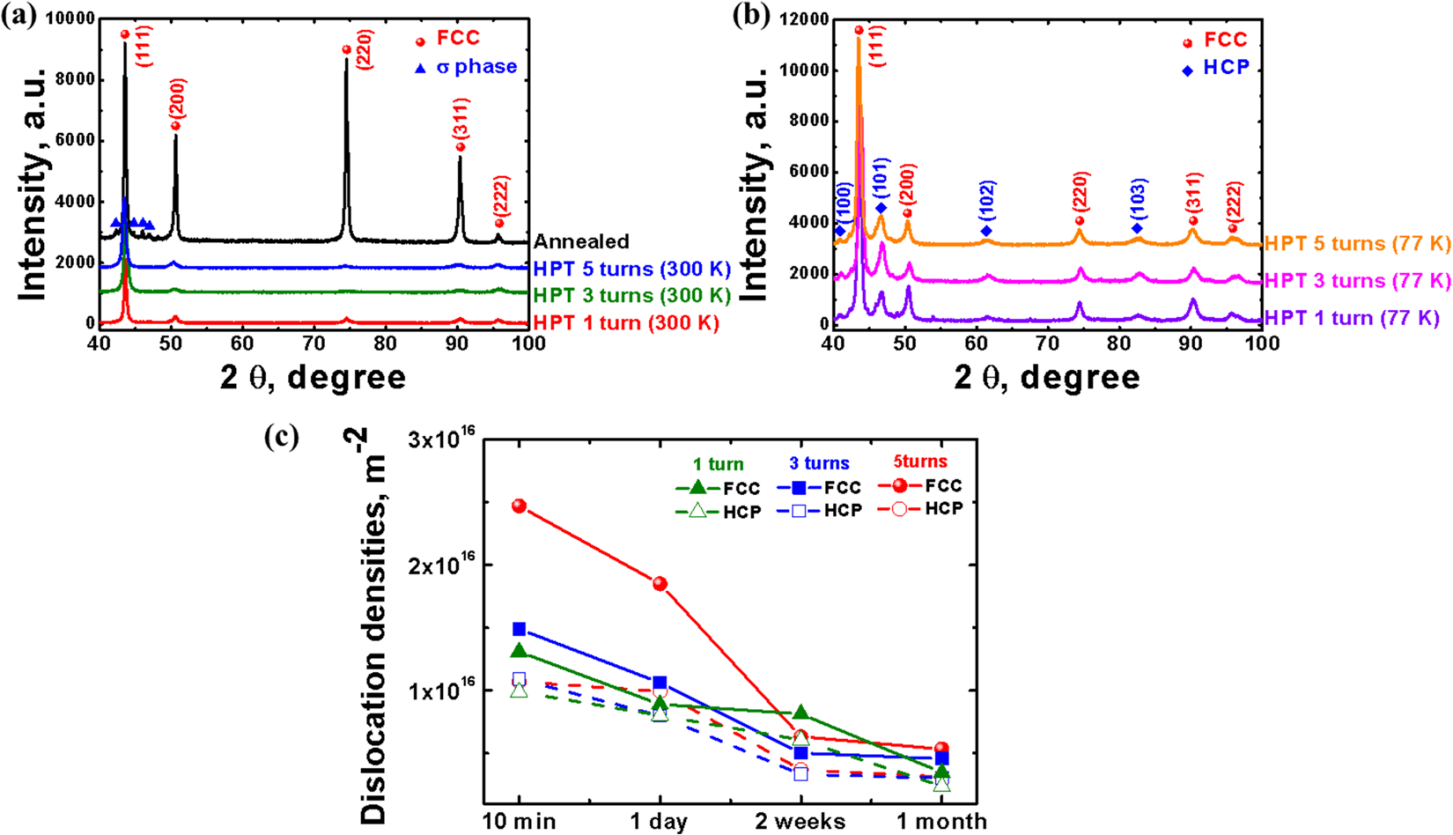
X-ray diffraction patterns and dislocation density evolution of Co_20_Cr_26_Fe_20_Mn_20_Ni_14_ alloy. X-ray diffraction patterns of alloy Co_20_Cr_26_Fe_20_Mn_20_Ni_14_ in the annealed state and after HPT by 1, 3, and 5 anvil turns conducted (**a**) at 300 K and (**b**) at 77 K. The peak intensity is shown in arbitrary units. (**c**) Dislocation density evolution with natural aging time measured by the CMWP method after processing by cryo-HPT.

Figure 7(a,b) show X-ray diffraction (XRD) patterns of alloy Co_20_Cr_26_Fe_20_Mn_20_Ni_14_ in the annealed state and after HPT by 1, 3, and 5 anvil turns at 300 K and 77 K. The XRD results for the annealed Co_20_Cr_26_Fe_20_Mn_20_Ni_14_ HEA reveal FCC peaks with additional minor peaks around the FCC (111) peak. Based on the work by Pickering *et al*.^29^, the minor peaks can be attributed to the topologically close packed σ phase. Tsai *et al*.^30^ suggested a criterion for the formation of σ phase in Cr- and/or V-containing HEAs using the average valence electron concentration (VEC). The Co_20_Cr_26_Fe_20_Mn_20_Ni_14_ HEA has a VEC of 7.76, which falls in the VEC range (6.88–7.84) favoring σ phase formation. However, the low intensity of the σ phase peaks suggests that the alloy is mainly comprised by FCC phase.

The XRD peaks of alloy Co_20_Cr_26_Fe_20_Mn_20_Ni_14_ processed by 1, 3, and 5 revolutions of the HPT anvil were lower and broader than those of the as-annealed sample. This is associated with peak broadening caused by grain refinement and residual microstrains. It is evident that the XRD peaks of the σ phase almost disappeared as a result of HPT processing at 77 K and 300 K, while most of the FCC peaks sustained this deformation. As shown in Fig. 7(b), the XRD peaks of the HCP structure were observed after HPT by 1, 3, and 5 anvil revolutions at 77 K. This confirms that a deformation-induced phase transformation occurred in accordance with the previous result^28^.

By combining the XRD (Fig. 7(a)), SADP (Fig. 6), fast Fourier transform (FFT), and atomic resolution TEM data (see Supplementary Figs S1–S3), the lattice parameters of the observed HCP structure, a and c, were calculated to be 0.2536 nm and 0.4116 nm, respectively. These values are consistent with those reported by Zhang *et al*.^31^.

Line profile analysis of the obtained XRD data to measure the variation of the dislocation density of the cryo-HPT specimens with natural aging time was performed using the convolutional multiple whole profile (CMWP) program^32,33^. All parameters used for the CMWP analysis are given in Supplementary Table S2. The values of the lattice parameters and the Burgers vectors of FCC and HCP structures were calculated using the XRD data in Fig. 7(a,b), while the average contrast factors of dislocations, ${\bar{C}}_{h00}$ and ${\bar{C}}_{{hk}0}$, were calculated using atomistic simulation based on the second nearest-neighbor modified embedded-atom method (2NN MEAM)^34^. Figure 7(c) shows the dislocation densities measured using the CMWP method as a function of natural aging time after processing by cryo-HPT. Natural aging for 10 min after the cryo-HPT process resulted in a very high dislocation density (~1.0 to 2.5 × 10^16^ m^−2^) in both FCC and HCP phases. Decrease of the dislocation density with natural aging time was observed for all numbers of anvil revolutions of cryo-HPT. It is noted that the dislocation density in the FCC phase is higher than that in the HCP phase.

## Discussion

According to previous studies^4,5,35–37^, strain hardening of HEAs of the CoCrFeMnNi system is governed by dislocation glide for room temperature deformation, whereas at cryogenic temperatures it is affected by both dislocation glide and nanoscale twinning. Low temperature activity of twinning usually is associated with an increase of the deforming stresses at low temperatures due to thermally activated type of plastic deformation^38^. However, the present results for alloy Co_20_Cr_26_Fe_20_Mn_20_Ni_14_ show an opposite trend.

Low room-temperature strength characteristics of cryo-HPT processed materials^39^, referred to as ‘self-annealing’^39–41^, were observed for pure metals. Edalati *et al*.^39^ reported this unusual softening and grain coarsening phenomenon in terms of natural aging after cryo-HPT, i.e., self-annealing due to static recrystallization to relieve the high stored energy imposed by cryo-HPT. It was suggested that the following factors cause a metal to be prone to self-annealing: (i) low melting temperature promoting higher mobility of dislocations, (ii) low stacking fault energy leading to increased stored energy of deformation, and (iii) low HPT processing temperature giving rise to increased applied stress. The current HEA system has a moderate melting temperature (1284 °C) compared with the melting temperatures of the metals considered by Edalati *et al*.^39^. It also has the lowest stacking fault energy (3.5 mJ/m^2^)^15^ among the HEAs of the CoCrFeMnNi system. Thus, it is to be expected that it may exhibit self-annealing, especially in the case of cryo-HPT. In addition, in the present work, abnormal softening effect and hardness degradation with natural aging was observed in the mechanical properties of alloy processed by cryo-HPT. To establish whether such self-annealing does apply to the current HEA system, electron microscopy studies by SEM and TEM, as well as dislocation density measurements using the CMWP analysis, were performed.

Studies have shown that FCC alloys with a medium magnitude of the SFE, typically from 18 to 45 mJ/m^2^, are more likely to form deformation twins, while those with a low SFE below 18 μmJ/m^2^ have the propensity to an FCC to HCP phase transformation^42^. This may be rationalized using an analogy with the low SFE austenitic steels, for which twinning is associated with the formation of *intrinsic* stacking faults^43^. As this process is inhibited at low deformation temperatures and for low SFE^44^, the occurrence of deformation twinning was not very pronounced in cryo-HPT of the HEA considered. Still, the lamellar twin structure found may have contributed to the coplanar slip character of the deformation of the grains under cryo-HPT. There is also an alternative explanation for the low volume fraction of twins observed in this condition. According to Gu *et al*.^45^, in conventional alloys plastic strain can be localized in micro shear bands. Should this apply to HEA alloys as well, destruction of deformation twins by these shear bands intersecting them might be a possible reason why no evidence of pronounced deformation twinning was found in the present case^46^.

The occurrence of nano-grains with random crystallograhpic orientations in the alloy processed by room temperature HPT can be considered in terms of the concepts broadly accepted for alloys processed by SPD^47–50^. Despite the low SFE of the HEA studied, twinning was obviously not the predominant deformation mode under severe plastic deformation by 300 K HPT. Rather, plastic deformation was furnished by dislocation slip, leading to the formation of subgrains. The observed increase of strength and decline of ductility with growing number of HPT turns (from 1 revolution to 3 and 5 revolutions) at 300 K reported above can be rationalized in terms of the grain refinement and increased dislocation density.

A remarkable finding is that the overall grain refinement effect – unlike in the case of room temperature HPT – was not significant after processing by cryo-HPT. This is in line with the lower mechanical strength observed after cryo-HPT and can be viewed as a result of “self-annealing” of the grain structure during natural aging that occurred over the time between the formation of the cryo-HPT induced grain structure and the electron microscopy measurements. This time was typically of the order of two months. The observed slip pattern in the cryo-HPT processed alloy is associated with the low SFE of this HEA possesses. It gives rise to a large distance between partial dislocations into which perfect dislocations dissociate in their glide planes, thus inhibiting cross-slip and causing the dislocations to arrange in planar arrays. The LAGBs observed in the EBSD are believed to have formed by dynamic recovery processes during cryo-HPT as the formation of Taylor lattice and microbands.

The exact mechanisms of grain refinement by severe plastic deformation are not entirely understood. One possible scenario is the formation of a dislocation cell structure that gradually transforms to a new fine grain structure through the accumulation of misorientation between the neighboring cells^47^. Moreover, according to Hughes^51^ and Park *et al*.^52,53^, intersection of microbands that develop at large strains may cause grain subdivision, thus giving rise to ultrafine and even nano-sized grains. For the nano-grains found in the alloy processed by 300 K HPT, it is evident that they were not formed by intersection of microbands, but are rather a result of dynamic recrystallization that originated from dislocation cell structures or Taylor lattice domains and gradually transformed to fine grain structures. Another possible grain refinement mechanism is the formation of microbands at Tayor lattice boundaries and their subdivision is a further grain refinement mechanism. It is thus quite possible that for the HEA considered, both mechanisms may be activated during cryo-HPT.

The occurrence of an HPT-induced FCC to HCP phase transformation may be rationalized using an analogy with the mechanism proposed by Talonen and Hänninen for low SFE stainless steel^46^. According to these authors, overlapping of stacking faults on {111} planes in an FCC austenitic steel during plastic deformation leads to the formation of shear bands or micro-shear bands. When the overlapping develops in a regular fashion on every second {111} plane, the HCP crystal structure forms. In case the overlapping is irregular, stacking fault bundles would form.

The observed phase transformation under cryo-HPT is also consistent with the recent findings of Zhang *et al*.^31^ who reported a polymorphic transition from FCC to HCP in an equiatomic CoCrFeMnNi alloy. They made a point that despite the high mixing entropy of an equiatomic HEA system, high-pressure and/or low temperature can help overcoming the energy barrier between its HCP and FCC polymorphs^31^. It can be conjectured that similar considerations apply for our Co_20_Cr_26_Fe_20_Mn_20_Ni_14_ alloy, in which the cryo-HPT makes a transition from the FCC phase to a thermodynamically more favourable HCP phase kinetically possible. The structural changes from co-existing FCC and σ phases to a sole FCC phase by room temperature HPT, or to HCP phase by cryo-HPT, are expected to affect the mechanical properties of the Co_20_Cr_26_Fe_20_Mn_20_Ni_14_ alloy studied.

Edalati *et al*.^39^ argued that self-annealing is hardly detectable by microstructural analysis because it occurs very rapidly. An alternative way to confirm the self-annealing phenomenon is by measuring the variation of the dislocation density with natural aging time by means of the CMWP analysis, which was employed in the present work.

The large dislocation densities are consistent with those obtained in a recent study by Heczel *et al*. (1.94 × 10^16^ m^−2^ after two HPT turns)^54^. It can be argued that the high concentration of the different alloying elements and the low SFE (3.5 mJ/m^2^) are the reason for the very high dislocation density found.

The observed decrease of the dislocation density with natural aging time for all numbers of anvil revolutions of cryo-HPT is seen as a clear indication of self-annealing. This trend is further supported by a drop of hardness. It is noted that the dislocation density in the FCC phase is higher than that in the HCP phase. This observation can be rationalized in terms of dislocation emission from phase boundaries into the FCC phase^55^ during deformation-induced phase transformation.

**Figure 8:**
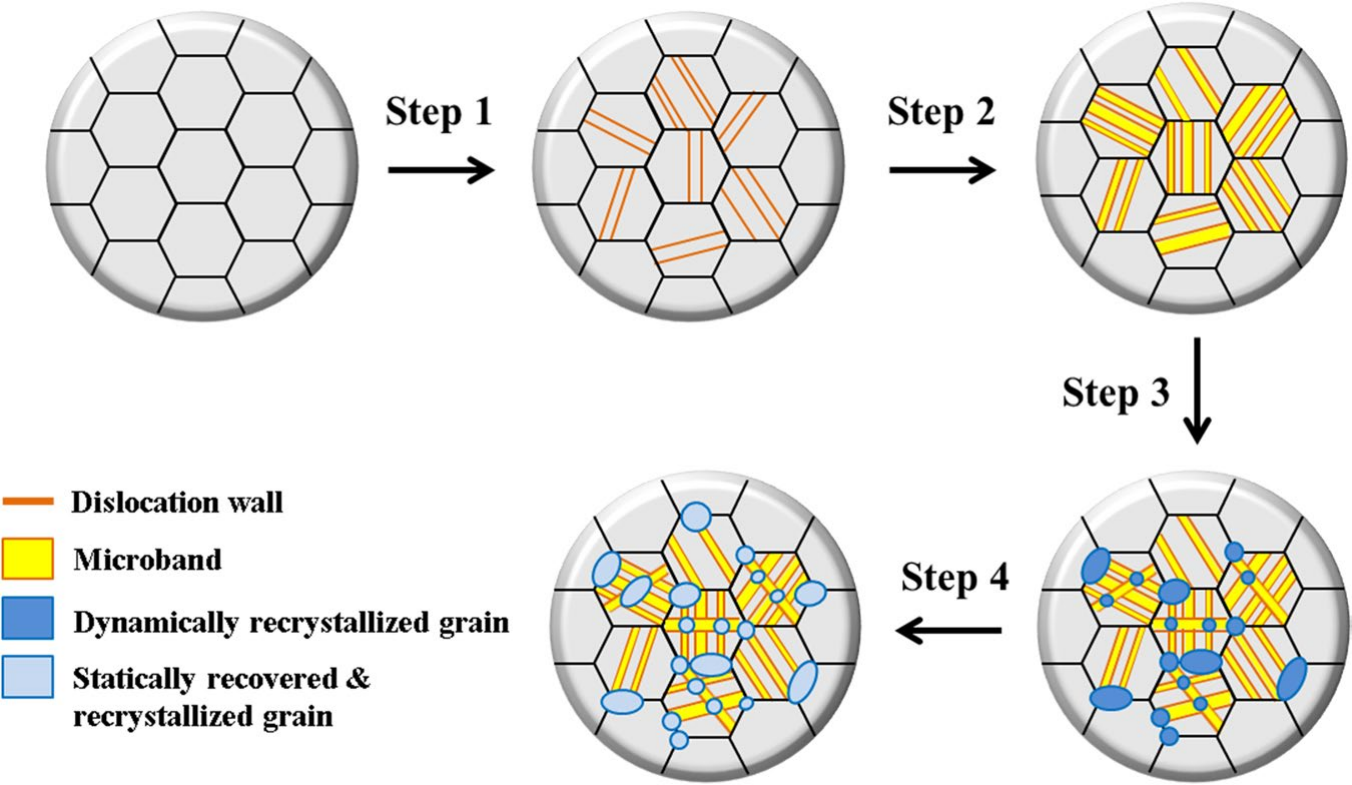
A schematic diagram illustrating the microstructural evolution of Co_20_Cr_26_Fe_20_Mn_20_Ni_14_ alloy. The microstructural evolution of Co_20_Cr_26_Fe_20_Mn_20_Ni_14_ alloy which accommodates the stored strain energy during and after cryo-HPT.

The results on microstructure and dislocation density evolution driven by the tendency of the Co_20_Cr_26_Fe_20_Mn_20_Ni_14_ alloy to accommodate the stored strain energy during and after cryo-HPT are summarized schematically in Fig. 8. The microstructural evolution is subdivided into four steps: first, dislocation rearrangement occurs by the formation of a Taylor lattice at the early stage of cryo-HPT (step I, Fig. 3(d)); with increasing strain, the Taylor lattice structure develops into a Taylor lattice domain with a single dislocation wall consisting of geometrically necessary dislocations. An energetically favorable structure is then formed through the formation of a second dislocation wall parallel to the first one, giving rise to a microband^56^ (step II, Fig. 3(e,f)). With further straining, grain refinement occurs by intersection of microbands and dynamic recrystallization (step III, Fig. 2(e)). Finally, with increasing natural aging time, static recovery and static recrystallization occur. This leads to a reduction of the dislocation density and the attendant drop of hardness (step IV, Figs 1(b–d) and 3(c)). It should be noted that the fragmentation of twins is yet another possible mechanism for grain refinement. However, for the sake of clarity, this process was not included in Fig. 8.

The abnormal grain structure that emerges after the application of high strains by cryo-HPT can be explained by low SFE of the alloy. The low SFE inhibits cross-slip of screw dislocations and, as a result, the mobility of dislocations decreases^57,58^. It is noted that low deformation temperature of cryo-HPT also lowers the SFE. The low mobility of dislocations in the HEA considered is also believed to suppress grain boundary migration – a process that is required for dynamic recrystallization to occur. Therefore, dynamic recovery processes such as the formation of a Taylor lattice and microbands prevailed over dynamic recrystallization during cryo-HPT. In addition, the rate of static recovery and self-annealing after cryo-HPT was slower in alloy Co_20_Cr_26_Fe_20_Mn_20_Ni_14_ than in pure copper^39–41^. It can be explained by the SFE difference between the present alloy and its pure constituents. The alloy in this study has the lower SFE than pure Cu. The SFE of Co_20_Cr_26_Fe_20_Mn_20_Ni_14_ alloy is 3.5 mJ/m^2^^15^, while that of pure Cu is 45 mJ/m^2^ ^39^. As mentioned above, low SFE-alloys have a low mobility of dislocations. Therefore, static recovery and static recrystallization, which may be conjectured to be the main processes underlying self-annealing, are sluggish during natural aging of alloy Co_20_Cr_26_Fe_20_Mn_20_Ni_14_.

## Conclusions

- It was established that room temperature and cryogenic HPT processing of Co_20_Cr_26_Fe_20_Mn_20_Ni_14_ had an effect on its microstructure and the attendant mechanical properties. However, the consequences of room temperature HPT and cryo-HPT for microstructure and mechanical properties were distinctly different. Thus, unlike in the case of room temperature HPT, a coarse grained structure supposedly stemming from static recovery co-existing with recrystallized grains was observed in the cryo-HPT processed alloy. This is consistent with the lower tensile strength, Vickers hardness, and dislocation density found after cryo-HPT.
- This surprising result may suggest that extreme grain refinement was hindered by the FCC to HCP phase transformation in the course of cryo-HPT. However, the occurrence of extreme grain refinement during the cryo-HPT process cannot be ruled out. Indeed, if nano-grained structures were produced *in situ*, they might have been unstable at room temperature and underwent recovery process before the electron microscopy characterization and the mechanical testing were done.
- The picture of the microstructural evolution leading to relaxation of the stored strain energy during and after cryo-HPT that was proposed based on the analysis of the experimental data obtained is as follows. The process is considered to occur in four major steps. First, under cryo-HPT, a Taylor lattice is formed, followed by the emergence of microbands and dynamically recrystallized grains. After processing by cryo-HPT, highly deformed grains undergo static recovery and self-annealing progressing with natural aging time.

## Materials and Methods

### Materials and high-pressure torsion

The annealed Co_20_Cr_26_Fe_20_Mn_20_Ni_14_ alloy was prepared by means of the method using in the previous work^28^. The HPT of the samples from the annealed HEA plate (discs with diameter of 10 mm and thickness of 1 mm) was performed at a cryogenic temperature (77 K) in liquid nitrogen and at room temperature (300 K) in air by N = 1, 3, and 5 revolutions of the HPT anvil under a pressure of 5 GPa normal to the disc-shaped sample. A thermocouple was located 2.5 mm away from the center of the disc to confirm that the temperature reached the steady-state level of 77 K during the HPT process. This site of the thermocouple corresponds to the location at which specimens for tensile testing were extracted from a disc. No slippage in processing by HPT was confirmed and the method in detail is presented in Supplementary Material.

### X-ray diffraction and dislocation density measurements

For phase identification, XRD was conducted before and after the HPT processing. The surfaces of samples for the XRD measurement were first polished using 600, 800, and 1200 SiC grit papers, then fine polished by 1 μm diamond powders in order to eliminate surface roughness. Rigaku D/MAX-2500 XRD equipment was used with the incident beam of Cu Kα radiation (wavelength = 1.5418 Å). The scans were performed from 40 to 100° of 2θ with a step size of 0.02° and a scan speed of 1°/min. Line profile analysis of the obtained XRD data to calculate dislocation densities of the cryo-HPT specimens was performed using the CMWP program^32,33^. XRD measurements for the CMWP analysis were carried out over time after processing cryo-HPT.

### Vickers hardness and tensile testing

Following the HPT process, the Vickers hardness was measured at the surface of the disc at various locations, from the center to the edge, 10 minutes, one day, two weeks, one month, and two months after HPT. Ahead of the Vickers hardness measurements, samples were polished using a 1200 SiC grit paper. The hardness was measured using an FM-700 microhardness tester with a load of 300 g and a dwell time of 10 s. Tensile testing was conducted with a strain rate of 10^−3^ s^−1^ on miniaturized specimens extracted from the HPT-processed discs. Dog-bone shaped samples with a gauge length of 1.5 mm and a width of 1 mm were used for tensile testing. Tensile tests for each condition corresponding to a particular number of HPT revolutions were conducted in triplicate to ensure reproducibility of results. During the tensile tests on miniaturized specimens, the strain measurements were done by a digital image correlation (DIC) method^59^. The specimens for electron microscopy characterization were prepared two months after they had been HPT processed.

### Microstructure characterization

SEM characterization was performed using a JEOL 7001F and a Philips XL30S devices. BSE images based on the diffraction contrast were taken to identify the evolution of grains and twins for the different processing schedules. EBSD measurements were performed using AztecHKL and TSL/OIM software. The data obtained were analyzed using HKL/Channel 5 and TSL/OIM. Texture analysis was conducted using JTEX^60^. SEM samples were prepared by grinding with SiC paper up to 2400-grit, followed by polishing with a colloidal silica suspension for 2 h. EBSD samples were subsequently polished by argon ion beam using a GATAN precision etching-coating system to remove the strained layer. TEM characterization was carried out using an FEI Tecnai F20 FEG TEM operating at an acceleration voltage of 200 kV. TEM foils were prepared by focused ion beam (FIB) lift-out performed on an FEI Quanta 3D FEG SEM. The area of the SEM and EBSD observation was 3 mm away from the center of the specimen. TEM foils were lifted out at this location, as well.

## Electronic supplementary material

Supplementary Information
