# Supplementary material for: Microstructure and Mechanical Properties of High-Entropy Alloy Co20Cr26Fe20Mn20Ni14 Processed by High-Pressure Torsion at 77 K and 300 K
Source: Sci Rep. 2018 Jul 23;8:11074. doi: 10.1038/s41598-018-29446-y (PMC6056509; doi:10.1038/s41598-018-29446-y)
Supplement: Supplementary file 1 — Supplementary Information [file 41598_2018_29446_MOESM1_ESM.docx]

Supplementary Information for

Microstructure and Mechanical Properties of High-Entropy Alloy Co_20_Cr_26_Fe_20_Mn_20_Ni_14_ Processed by High-Pressure Torsion at 77 K and 300 K

Jongun Moon^a,b^, Yuanshen Qi^c^, Elena Tabachnikova^d^, Yuri Estrin^e,f^, Won-Mi Choi^a^, Soo-Hyun Joo^g^, Byeong-Joo Lee^a^, Aleksey Podolskiy^d^, Mikhail Tikhonovsky^h^, and Hyoung Seop Kim^a,b*^

^a^ Department of Materials Science and Engineering, Pohang University of Science and Technology (POSTECH), Pohang 790-784, Korea

^b^ Center for High Entropy Alloys, Pohang University of Science and Technology (POSTECH), Pohang 790-784, Korea

^c^ Department of Materials Science and Engineering, Technion – Israel Institute of Technology, 32000, Haifa, Israel

*^d^ B. Verkin Institute for Low Temperature Physics and Engineering of National Academy of Sciences of Ukraine, 47 Nauky Ave., Kharkov, 61103, Ukraine*

*^e^ Department of Materials Science and Engineering, Monash University, Melbourne VIC, 3800, Australia*

*^f^ Department of Mechanical Engineering, The University of Western Australia, Crawley, WA6009, Australia*

^g^ Institute of Materials Research, Tohoku University, Sendai 980-8577, Japan

*^h^ National Science Center «Kharkov Institute of Physics and Technology» of National Academy of Sciences of Ukraine, 1 Academicheskaya street, Kharkov, 61108, Ukraine*

**Deformation-induced phase transformation after the HPT at 77 K**

Beside a partial area of a grain that has gone through the phase transformation, a complete grain with the hexagonal-closed packed (HCP) crystal structure can also be found. Highlighted by a box labeled “b” in Fig. S1(a) and presented with a lattice image in Fig. S1(b), a nano-grain with an HCP crystal structure, around 30 nm in size, is situated at the boundary between two bigger grains. The grain located above this nano-grain is on its [011] zone axis as indicated by the fast Fourier transform (FFT) image shown in Fig. S1(c). Figure S1(d) shows that the nano-grain is on its $[2\bar{1}\bar{1}0]$ zone axis. The [011] direction of FCC structure is nearly parallel to the [0002] direction of HCP structure with an angle of 13º between them, as indicated in Fig. S1(e). Figure S1(f) shows the FFT image of the lattice beneath the nano-grain. This area has transformed to HCP as well, its crystallographic orientation being 90º away from that of the nano-grain. It can be conjectured that the nano-grain was generated by the interaction between the face-centered cubic (FCC) and HCP grains, represented by the regions (c) and (f) in Fig. S1(b).

Figures S2(a–c) show two neighboring grains with different activated slip directions. From Figs. S2(d, f), it can be established that some parts of the grains have fully transformed to HCP structure as revealed by their FFT images. Also, the inverse FFT (IFFT) images shown in Figs. S2(g, i) confirm the HCP structure by indicating the ABABAB stacking sequence along the [0001] direction. Figure S2(e) shows a region with FCC structure near the boundary. The ABCABC stacking sequence along the [111] direction in this region is presented in Fig. S2(h). The angle between the [0001] direction in Fig. S2(g) and the [111] direction in Fig. S2(h) is around 17º.

Figure S3 provides a more detailed view of the transition zone from the FCC to the HCP regions. Figure S3(b) displays a transition zone between the FCC and the HCP regions, which is represented by a dense bundle of stacking faults. This transition zone can be regarded as a site of nucleation of deformation-induced phase transformation.

**Slippage in processing by HPT at 300 K and 77 K**

To confirm no slippage occurred during HPT, we inserted copper along the radial direction to mark the HPT disk as shown in Fig. S4(a). Then, the copper-inserted disk was processed by one revolution of the HPT under a pressure of 5 GPa at 300 K and 77 K. If a single revolution of the HPT rotates properly without the slippage, the position of copper will be the same when the disk was flipped upside down. Figures S4(b, c) and S4(d, e) show the copper-inserted disks after a turn of HPT processing at 300 K. The images of back side were obtained by flipping the disk along the direction perpendicular with respect to the direction where copper inserted. The vertical cross-section of the HPT processed disk at 77 K shows copper has a torsional flow starting from the surface as shonw in Fig. S4(f). No significant slippage which could affect the mechanical porperties and microstructures of the HPT processed alloy at 300 K and 77 K was observed.

**Supplementary Figures**

**
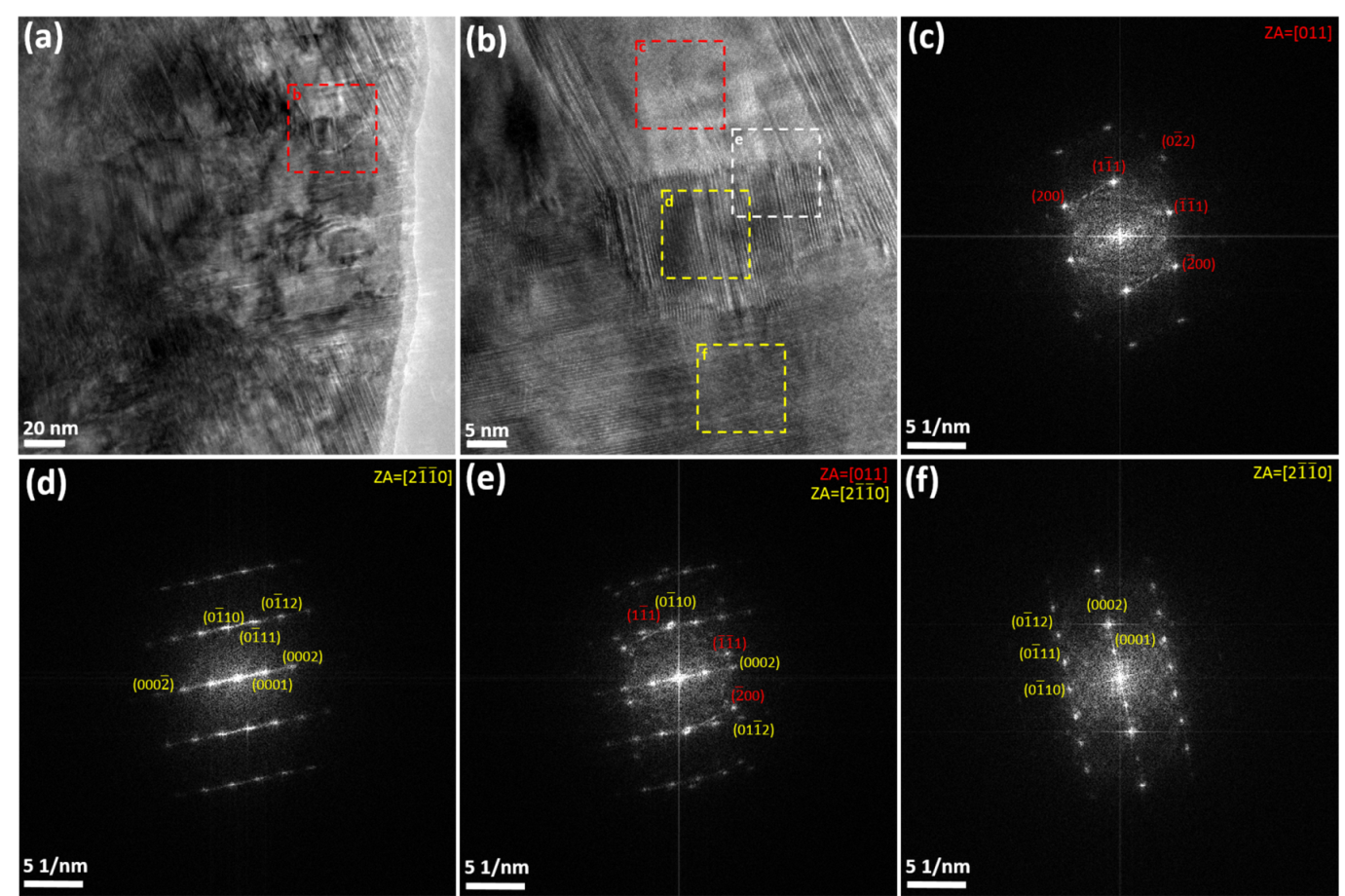
**

**Figure S1. Nano-grain with HCP crystal structure.**

(a) TEM image of Co_20_Cr_26_Fe_20_Mn_20_Ni_14_ alloy after 5 turns of the HPT at 77 K showing a nano-grain with HCP crystal structure (marked by a red box and labeled ‘b’); (b) a lattice image of the nano-grain; (c-f) FFT images of the regions selected from (b).

**
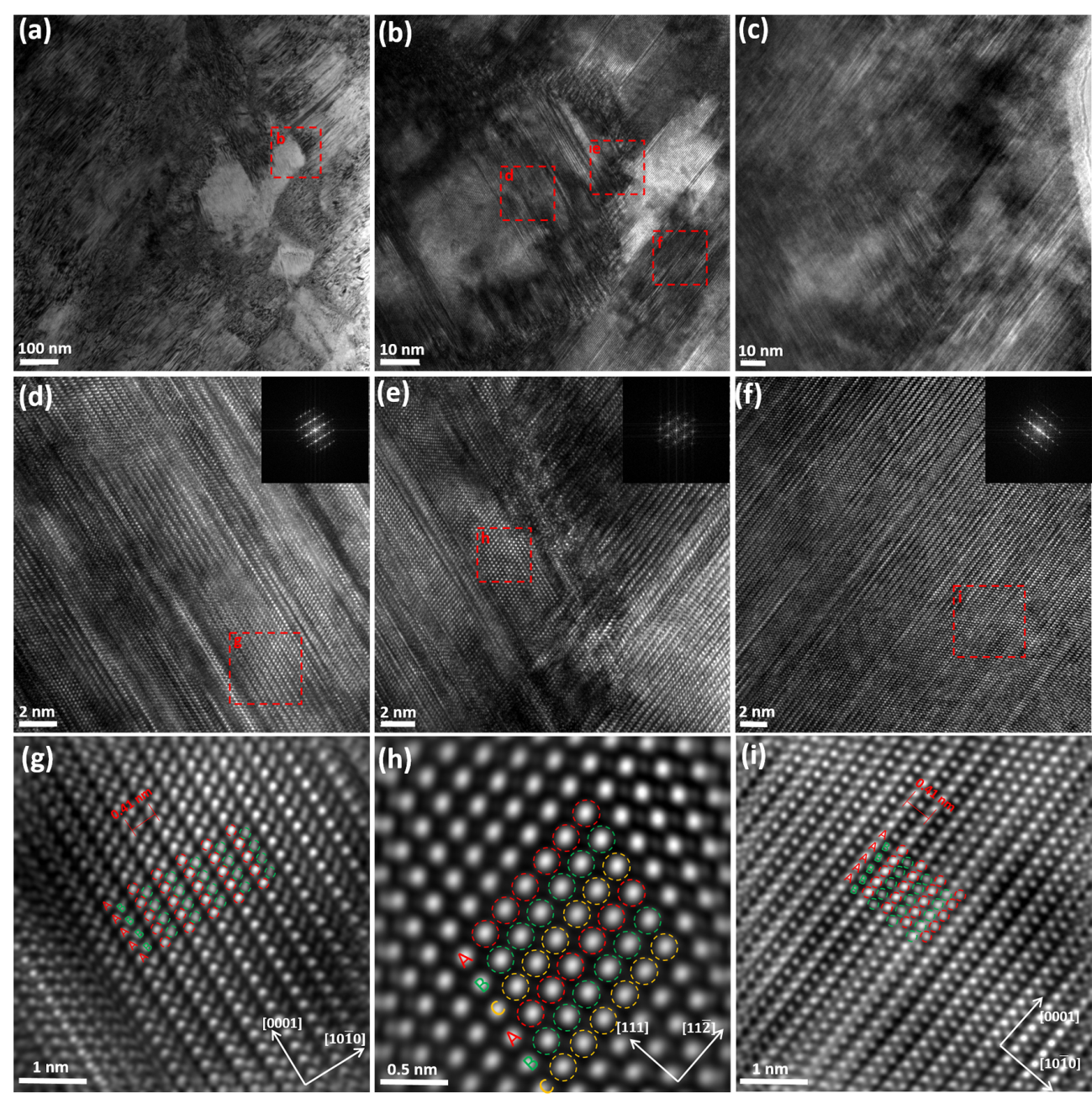
 Figure S2. Micrographs of Co_20_Cr_26_Fe_20_Mn_20_Ni_14_ alloy after 5 turns of the HPT at 77 K.**

(a, b) Bright field TEM (BF-TEM) images; (c) TEM images; (d-f) high resolution TEM (HR-TEM) images (d-f) taken from the regions highlighted in (b); (g-i) atomic-resolution TEM images taken from the regions marked in (d-f), respectively. Insets in (d-f) are the FFT images of (g-i), respectively.

**
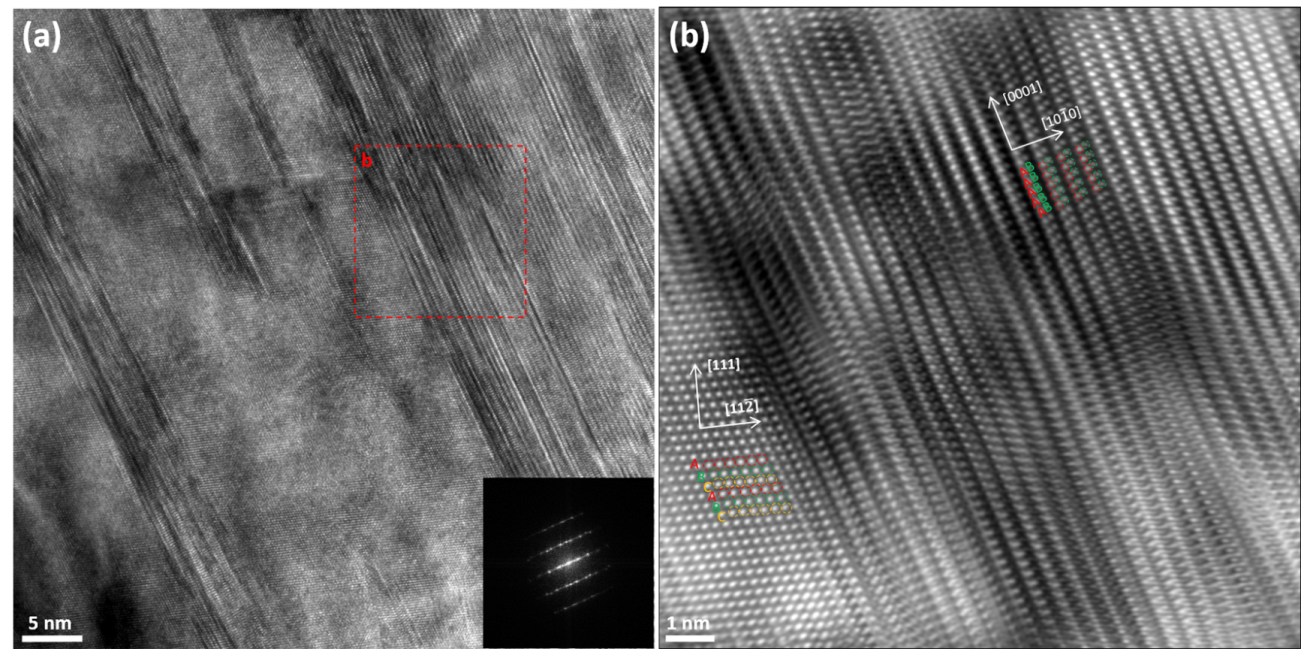
**

**Figure S3. Phase transformation from FCC to HCP.**

(a) HR-TEM and (b) atomic-resolution TEM images showing a transition from FCC to HCP crystal structure as a result of 5 HPT turns at 77 K. The inset in (a) represents the FFT of the region (b).


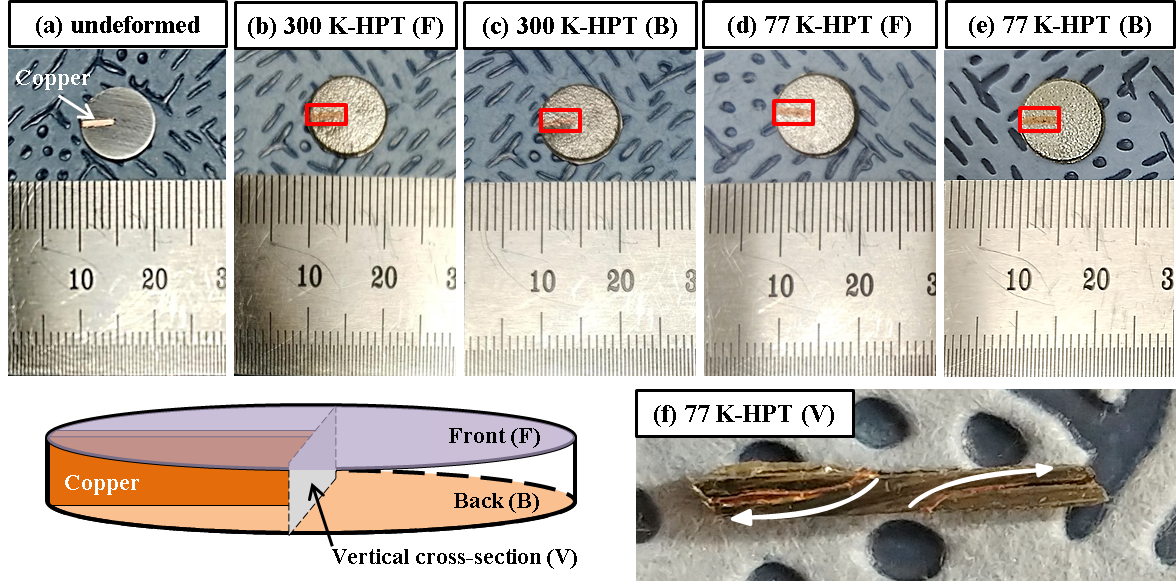


**Figure S4. Front, back sides and vertical cross-section of copper-inserted HPT disks after processing by HPT at 300 K and 77 K.**

(a) undeformed disk, (b, c) front (F) and back (B) sides of disk after processing HPT at 300 K, (d, e) front (F) and back (B) sides of disk after processing HPT at 77 K, and (f) vertical cross-section (V) of disk after processing HPT at 77 K. White arrows in (f) show copper flow after a revolution of HPT.

**Supplementary Tables**

**Table S1. Mechanical characteristics of Co_20_Cr_26_Fe_20_Mn_20_Ni_14_ alloy tested in tension at room temperature two weeks after HPT processing at 77 K and 300 K.**

| **Mechanical properties** | **HPT at 77 K** | | | | **HPT at 300 K** | | |
| --- | --- | --- | --- | --- | --- | --- | --- |
|  | **1 turn** | **3 turns** | **5 turns** | **1 turn** | | **3 turns** | **5 turns** |
| Yield strength, MPa | 985 | 1048 | 1116 | 1520 | | 1885 | 1837 |
| Tensile strength, MPa | 1150 | 1339 | 1543 | 1824 | | 2074 | 2060 |
| Elongation,  % | 13.9 | 5.6 | 4.6 | 9.2 | | 5.6 | 4.8 |

**Table S2. The parameter values used in the CMWP analysis.**

| Crystal structure | Lattice parameter, nm | | Burgers vector, nm | *_h00_* or *_hk0_* 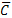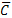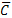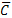 |
| --- | --- | --- | --- | --- |
|  | a | c |  |  |
| FCC | 0.3598 | - | 0.2449 | 0.1504 |
| HCP | 0.2536 | 0.4116 | 0.3845 | 0.1023 |
